# Study on the Mechanical Properties of Modified Sludge Soil Based on an SM-C Modifier

**DOI:** 10.3390/ma18030483

**Published:** 2025-01-21

**Authors:** Jun Nie, Kai Zhang, Xiangyang Fan, Yixuan Zhang, Guoxu Wei, Xiangyong Yu, Wen Xu

**Affiliations:** 1 Nanchang Expressway Co., Ltd., Nanchang 330009, China; 2 Jiangxi Transportation Institute Co., Ltd., Nanchang 330200, China; 3 Road Material and Structure Engineering Technology Research Center of Jiangxi Provincial, Nanchang 330200, China; 4 School of Transportation and Logistics Engineering, Wuhan University of Technology, Wuhan 430063, China

**Keywords:** modified sludge soil, mechanical properties, roadbed filling, triaxial test

## Abstract

The purpose of this study is to solve the problem of the harmless treatment of dredged silt and soil extraction during road construction in lake areas. The silt in the project area is used as the research material to evaluate its engineering applicability as an improved filling material for the roadbed of the lake’s surrounding road. Through indoor pretreatment and a series of mechanical performance tests, including compaction tests, unconfined compressive strength tests (UCS), bearing ratio tests (CBR), triaxial compression tests (CU consolidated undrained), and consolidation tests, we obtained key mechanical parameters of modified sludge soil, such as maximum dry density, optimal moisture content, unconfined compressive strength, bearing ratio, shear strength, and compression characteristics. The research results show that with the increase in modifier dosage, the optimal moisture content of modified sludge soil increases, the maximum dry density decreases, and its compressive strength and shear strength significantly improve. The CBR value also meets the technical requirements of each layer of the roadbed. Specifically, after 7 days of curing, the compaction degree of 10% modified sludge soil can exceed 96%, the unconfined compressive strength reaches 0.819 MPa, the CBR value reaches 17.5, the cohesion measured by triaxial tests is 78 kPa, the internal friction angle is 27°, and it exhibits low compressibility. These findings provide new solutions for environmentally friendly treatment, resource utilization, and road engineering in river and lake sediments.

## 1. Introduction

With the rapid development of China’s transportation network and the continuous increase in highway mileage and network density, the construction of transportation infrastructure in complex geological areas is increasing, bringing a series of technical challenges to road engineering [1].

In situations where the regional environment is complex and the special soil condition standards for road filling are difficult to meet, it is crucial to effectively handle and utilize local filling resources with poor performance in order to reduce road construction costs and alleviate material shortages. Among these special soils, silt and similar soils are particularly common, which usually have characteristics such as high compressibility, large porosity, high natural moisture content, and low shear strength [2]. Due to these characteristics, silt and silty soil cannot be directly used for engineering construction and must undergo modified solidification treatment. The interdisciplinary research between environmental science and civil engineering provides a new approach for the treatment and improvement of these special soils. This article investigates how to convert waste sludge into valuable geopolymer materials for road construction and explores the process of converting waste sludge into geopolymers, which may involve chemical reactions that react to the silicon aluminum components in sludge with alkaline solutions to generate geopolymers [3]. This study is based on the Luhu Environmental Remediation and Cultural Tourism Comprehensive Development Project in Jiangxia District, Wuhan City. The silt in the project area was selected as the research object, and the engineering applicability and mechanical properties of modified silt soil as a filling material for the road subgrade around the lake were systematically studied. The research results provide innovative ideas for the environmentally friendly treatment and resource utilization of river and lake sediments, as well as road engineering construction in the lake area, which is in line with the concept of sustainable development and has significant economic and social benefits.

Many researchers at home and abroad use multiple curing agent components to prepare composite curing agents and determine the curing agent formula using orthogonal experimental methods. Mud modified soil is used as roadbed filling material to verify its mechanical properties. The testing methods include a compaction test, unconfined compressive strength test, CBR test, shear strength test, and a compression test [4,5,6,7,8]. The main conclusions are as follows: (1) Compaction characteristics—different curing agents represent different mechanisms of action, so the compaction characteristics of modified soil also vary. The use of sulfur fixing ash and lime-modified sludge reduces the maximum dry density, increases the maximum moisture content, and requires greater compaction effort. (2) Compressive strength—the research content mainly includes the effect of intensity enhancement and influencing factors. Zhang Rongjun [9] proposed through experiments that moisture content and cement dosage have independent effects on each other, and obtained a strength prediction formula for low cement dosage. Other influencing factors include curing temperature [10], water cement ratio [11], minimum dosage [12], curing age, and the synergistic modification of other additives [13]. The influence of each factor on compressive strength was obtained through experiments, and the strength was predicted through empirical formulas. (3) As a requirement indicator in the specification, CBR mainly obtains influencing factors through experiments [14], including compaction degree, curing age, and curing agent. (4) Shear strength—the main analysis focuses on the stress–strain relationship and the variation law of failure strain, with shear strength parameters as the main detection indicators. Wang Dongxing [15] replaced cement low-calcium fly ash with lime low-calcium fly ash in the same proportion, and the stress–strain relationship of the specimen changed from plasticity to brittleness, with a significant reduction in failure strain. The effect of different material curing agents was compared at 360 days of strength. (5) Compression performance [16,17,18]—the compression performance of modified soil is evaluated based on the compression modulus and compression coefficient, and the effects of dosage, initial moisture content, and curing age on the compression performance are evaluated. Different compression models are used to represent its compression characteristics. Li et al. [19] studied the use of industrial by-products such as fly ash, soda ash residue, and flue gas desulfurization gypsum (FGD gypsum) combined with lime to improve the mechanical and microstructural properties of excavated soil, reduce the use of lime, and enhance the engineering performance of soil.

Based on the current research status at home and abroad, this article takes the Luhu silt in Jiangxia District, Wuhan City as the research object, modifies the collected silt soil samples based on an SM-C modifier, and conducts mechanical property tests on the modified sludge soil. By conducting compaction tests, unconfined compressive strength tests, CBR tests, triaxial tests, and consolidation tests, the changes in the mechanical properties of modified sludge soil materials under the influence of factors such as modifier dosage, curing age, compaction degree, and initial moisture content were studied. The main issues addressed in this study include the specific effects of SM-C amendments on the mechanical properties of silt soil, optimal dosage, and performance under different environmental conditions.

## 2. Materials and Methods

### 2.1. Materials

The soil sample was taken from Luhu Lake in Jiangxia District, Wuhan. The sampling point was close to the planned route of the scenic area road, and 3–4 sampling sites were selected with a sampling depth of about 1.0 m. The soil sample is gray-brown in color. The main physical property indexes of the soil sample are as follows: initial water content is 60 ± 5, wet density is 1.686 g·cm^−3^, dry density is 1.038 g·cm^−3^, specific gravity is 2.71, pore ratio is 1.62, plastic limit is 26%, liquid limit is 48.7%, and plastic index is 25%.

The modifier used in the experiment is an SM-C-type modifier from Guangzhou Zhengyuan Environmental Engineering Co., Ltd. (Guangzhou, China), and the product is in solid powder form. The modifier is composed of a certain proportion of various oxide components, mixed and reacted at a high temperature of 1200 °C. SM-C modifier is mostly used for sludge treatment. By treating the sludge with the inorganic composite material of ferrosilicon aluminum in the modifier, it overcomes the technical defect of the traditional curing agent, which can only increase the solid content in the mixture; it thoroughly modifies the sludge from the aspect of physical and chemical properties, breaks the colloidal structure of the mud, and converts the sludge into engineering raw materials with high engineering performance, which can be used as subgrade fillers. The modified soil can prevent secondary slime.

### 2.2. Compaction Test

This study used heavy compaction and wet sampling techniques to systematically investigate the effect of modifier dosage ranging from 4% to 15% on the compaction characteristics of modified sludge soil. In the experiment, six different mixing ratios of 0% (i.e., plain soil), 4%, 8%, 10%, 12%, and 15% were selected.

For plain soil samples, we first air dried them at a temperature not exceeding 80 °C, until the moisture content drops to near the plastic limit. Then, five samples with different moisture contents were prepared for compaction tests in increments of 2% each. For modified sludge soil, mixing and indoor curing are required first to achieve a moisture content close to the plastic limit, and the moisture content is also distributed with a difference of 2%.

**Figure 1:**
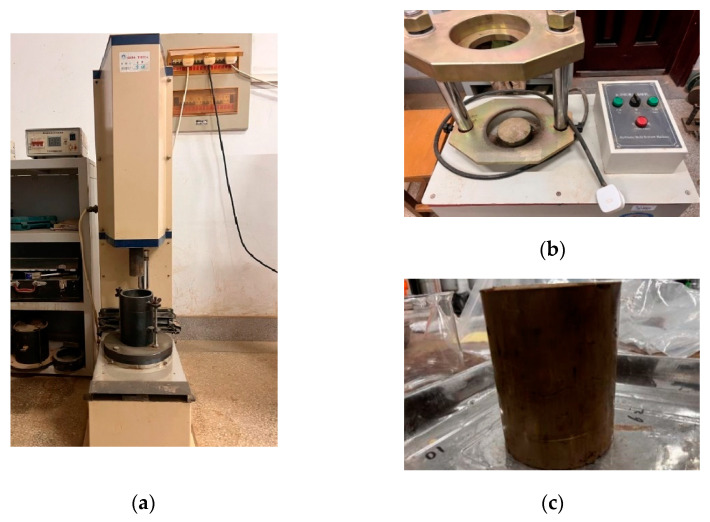
Compaction test instrument and specimen: (**a**) electric compaction device; (**b**) automatic film release device; (**c**) specimen.

The instruments used in the compaction test are shown in Figure 1, including the compaction device and automatic film release device. The inner diameter of the specimen is 10cm and the height is 12.7 cm. After compaction is completed, we first weighed the mass of the specimen and then cut a soil sample from the center of the specimen to determine the moisture content. The wet density of the compacted soil sample can be calculated by comparing the quality difference between the compacted specimen and the compacted cylinder. Based on the data of the moisture content sample, we calculated the dry density of the soil sample. These data provide an important basis for analyzing the compaction characteristics of modified sludge soil and its variation with dosage.

### 2.3. Unconfined Compressive Strength Test

#### 2.3.1. Design of Experimental Parameters for Modifier Dosage

This study designed three key experimental parameters in detail, including the dosage of modifier, initial moisture content, and curing age, to explore their effects on the unconfined compressive strength of modified sludge soil.

The dosage of modifier: The reaction between the modifier and certain components in the sludge is the key to strength formation, so the dosage is the main influencing factor. This experiment set dosages of 0%, 4%, 8%, 10%, 12%, and 15% to reveal the relationship between dosage and strength. Choosing a larger dosage is to explore the relationship between dosage and strength in indoor experiments. In practical engineering, the appropriate dosage should be selected according to specific requirements.

Initial moisture content: Even if the specimen is prepared according to the optimal moisture content, the initial moisture content still has an impact on the modified soil. This experiment set initial moisture contents of 50%, 65%, and 80% to separately study their effects on the properties of modified soil, while maintaining a constant dry soil mass.

Maintenance parameters: It is expected that the compressive strength of modified sludge soil will increase with the increase in maintenance age. This experiment selected curing ages of 1 day, 3 days, 7 days, 14 days, and 28 days to study the strength development of specimens in the early, middle, and stable stages of curing. Considering that the hydration reaction has already started in the pretreatment, a curing period of 1 day has been added to study the formation of initial strength. The maintenance conditions are set as indoor standard maintenance, with a temperature of 20 °C and humidity controlled at 90 ± 5% RH, slightly lower than outdoor summer temperatures, to simulate the effects of humidity and temperature on later strength.

#### 2.3.2. Experimental Plan Design

This study used two methods to prepare test specimens to evaluate the effects of modifier dosage and initial moisture content on the unconfined compressive strength of modified sludge soil.

Option 1: Without considering changes in initial moisture content, set the initial moisture content to 65% and conduct 5 different age periods (1 day, 3 days, 7 days, 14 days, and 28 days) of testing on specimens with 5 different levels of modifier dosage. Each specimen is subjected to two parallel tests, with a total of 10 specimens prepared. The test results are taken as the average of the two tests. Therefore, a total of 50 sets of specimens need to be prepared for 5 levels of modifier dosage.

Option 2: Investigate the effect of initial moisture content on later strength by conducting 28 day curing tests on specimens with 5 different dosage levels at three different initial moisture contents (50%, 65%, and 80%), and preparing a total of 20 sets of specimens.

**Figure 2:**
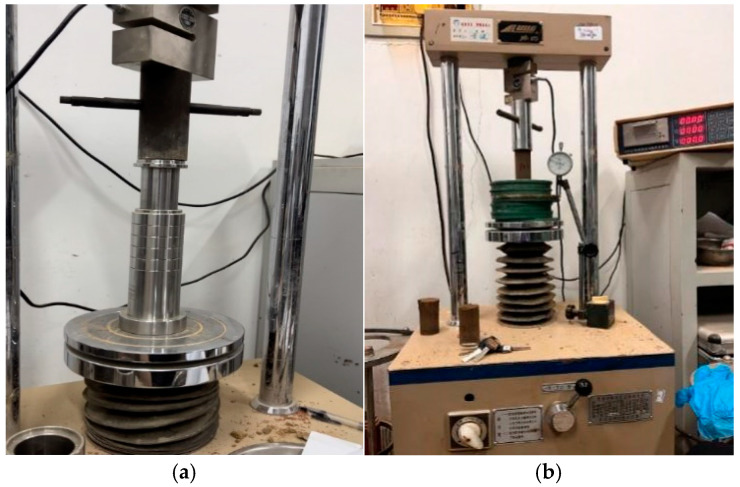
Sample preparation and unconfined compressive strength test: (**a**) preparation of specimen; (**b**) unconfined compression strength test.

**Figure 3:**
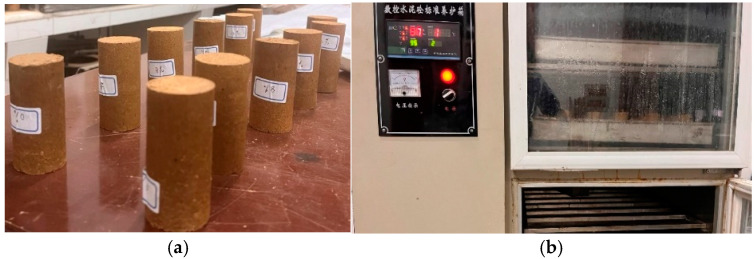
Preparation and curing of test specimen: (**a**) specimen; (**b**) curing process.

Taking into account both options, the total number of specimens for unconfined compressive strength testing is 70. The sample size is 3.91 cm × 8 cm, and the compaction degree was set to 95%. The material used for the soil sample was calculated based on the compaction degree and maximum dry density. The unconfined compressive strength test was prepared using the compression method, as shown in Figure 2a. The instrument used is a universal press, as shown in Figure 2b. The diameter of the pressure head is slightly larger than the specimen, and the sensor range is 30 kN, used to read longitudinal pressure. The displacement range was 30 mm, and a dial gauge was used for reading. The specimens were cured in a standard curing room, with temperature and humidity set to 20 °C and 90 ± 5% RH, respectively. The appearance of the specimen is shown in Figure 3a, and the curing box is shown in Figure 3b.

### 2.4. CBR Test

This study thoroughly investigated the effects of modifier dosage and compaction degree on the CBR value of modified sludge soil through CBR experiments. The test piece adopts a large compaction cylinder with an inner diameter of 152 mm and a height of 170mm, prepared by a heavy-duty compaction method. The compaction degree was set at three levels, 92%, 94%, and 96%, and the modifier dosage was selected at four levels, 4%, 8%, 10%, and 12%. The soil sample used was air-dried soil sieved through a 5 mm sieve, and each specimen underwent three parallel tests, totaling 36 sets of specimens.

**Figure 4:**
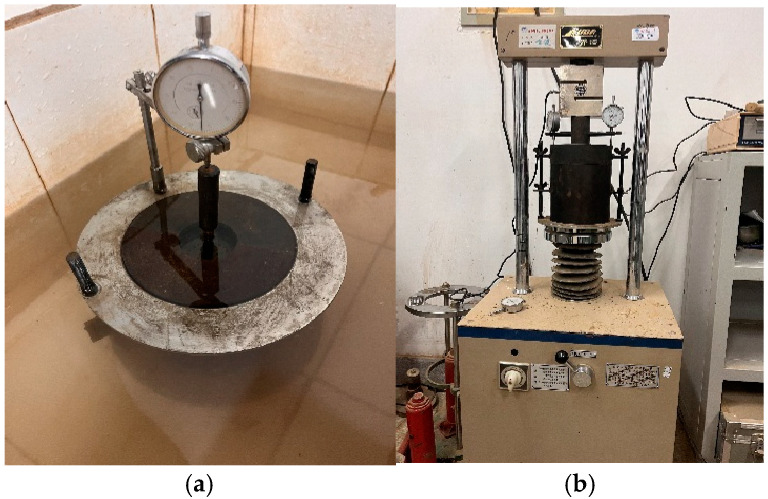
Schematic diagram of CBR test process: (**a**) immersion in water; (**b**) penetration test.

The experiment uses a pavement material strength meter, which was divided into two stages: immersion and penetration. During the immersion stage, the specimen was not demolded after compaction. The filter paper, porous plate, and load plate were placed in sequence, and a dial gauge was installed. The immersion time was 96 h. In the penetration test, a 7.5 kN force measuring ring was used, and penetration was carried out at a speed of 1mm/min. The vertical pressure was read by the sensor, and the displacement data on both sides were obtained through a dial gauge. The experimental process is detailed in Figure 4.

### 2.5. Triaxial Test

**Figure 5:**
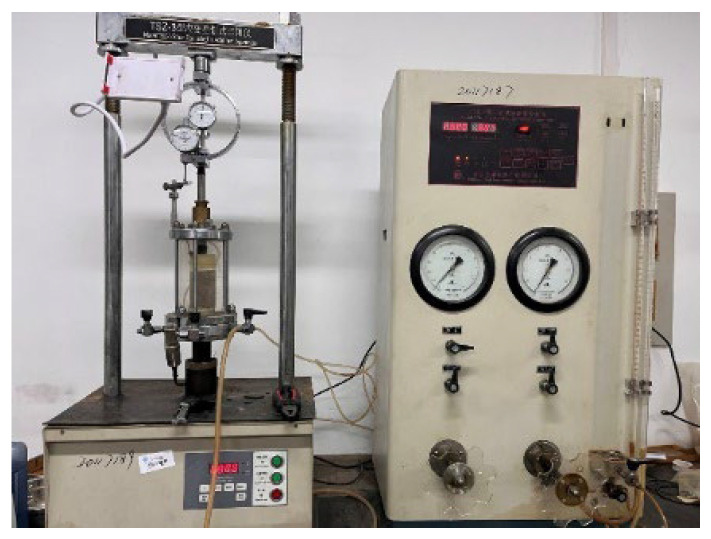
Triaxial testing instrument.

**Figure 6:**
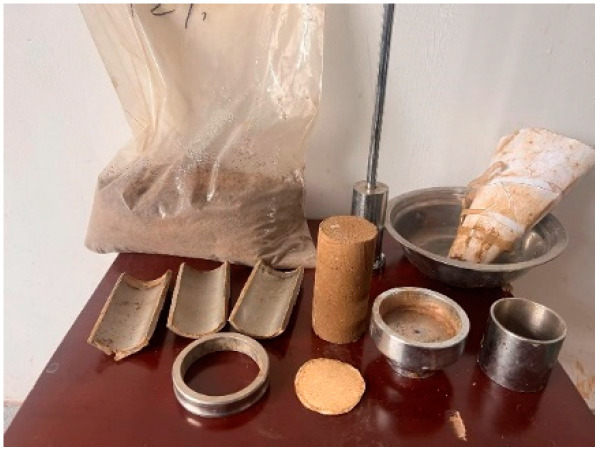
Triaxial specimens.

This study used consolidated undrained triaxial tests to analyze the stress–strain curve, failure mode, and shear strength parameters of modified sludge soil. The experiment used a strain gauge triaxial apparatus produced by Nanjing Soil Instrument Factory, as shown in Figure 5. The specimen preparation used the compactor shown in Figure 6, with a diameter of 39.1 mm and a height of 80 mm. The experiment selected 3 and 7 days as the curing age, and all specimens were subjected to saturation treatment. After reaching the curing age, the specimen was wrapped in a thin film and placed in a saturator for saturation using the suction method.

### 2.6. Consolidation Test

This article evaluates the compressive performance of modified sludge soil through standard consolidation tests, based on Terzaghi’s unidirectional consolidation theory, with a stability standard of settlement less than 0.01 mm within 1 h. The goal is to obtain indicators such as the compression coefficient, compression modulus, compression index, and consolidation coefficient, and analyze the effects of compaction degree, modifier dosage, and curing age on compression performance.

**Figure 7:**
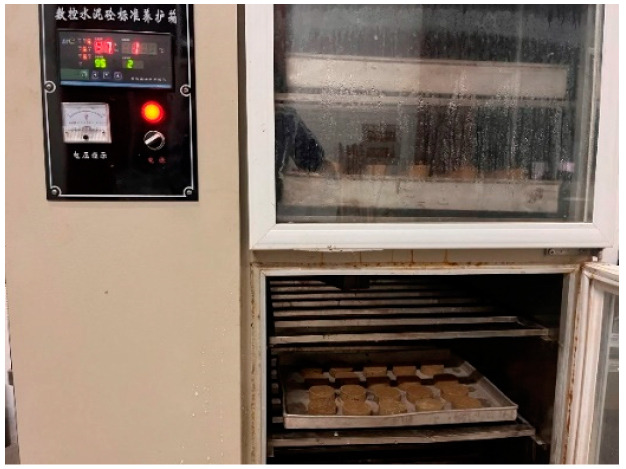
Curing of consolidation test specimens.

**Figure 8:**
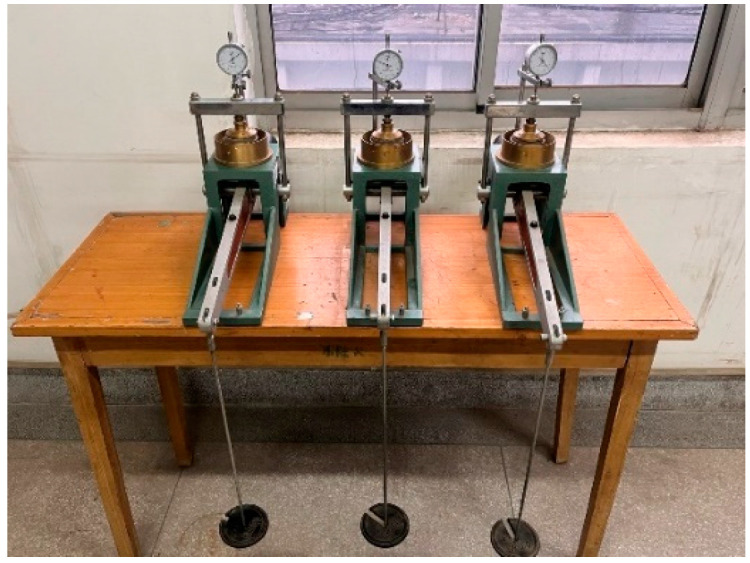
Consolidation test.

The specimens were prepared by the compression method, and the amount was calculated based on the compaction degree. They were cured in a standard curing environment until the specified age, as shown in Figure 7. Vacuum saturation was performed before the test. After installing the specimen, the consolidation instrument was adjusted to zero under preloading cell pressure, and the inner ring dial gauge was immediately loaded once it was greater than zero. The first level load was 50 kPa, and then the settlement rate and consolidation coefficient were measured according to the specifications. After 24 h of consolidation and stability, 100 kPa, 200 kPa, and 400 kPa loads were applied sequentially. The process is detailed in Figure 8. The number of specimens was 14, with dosages of 4%, 8%, 10%, 12%, and 15%. The compaction degrees for the 10% dosage were 92%, 94%, and 96%, respectively. The curing ages are 3 days and 7 days, respectively. The main consolidation performance indicators include the following: initial porosity ($e_{0}$), unit settlement ($S_{i}$), compression coefficient within a certain load range ($a_{v}$), compression modulus ($E_{s}$) within a certain load range, volumetric compression modulus ($m_{v}$), and compression index ($C_{c}$).

## 3. Results and Discussion

### 3.1. Results and Analysis of Compaction Test

**Figure 9:**
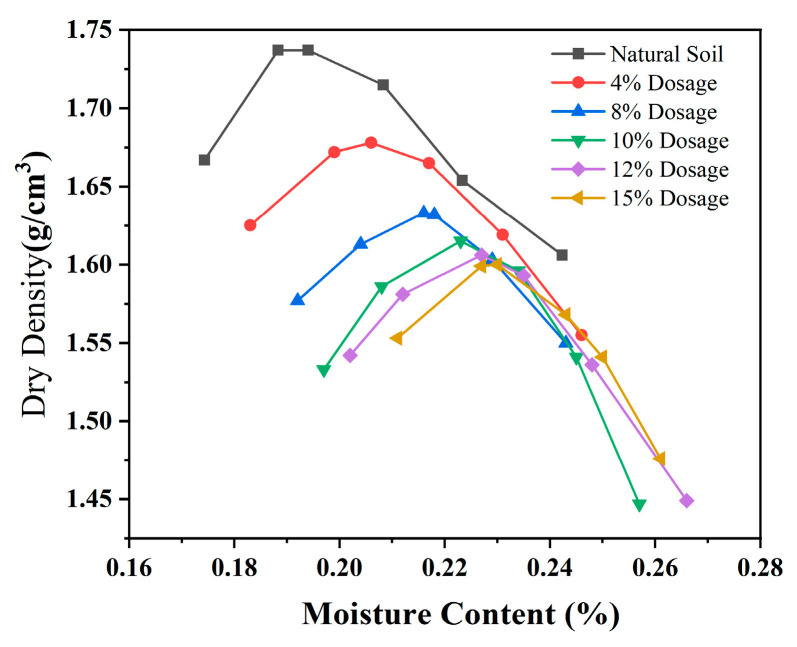
Compaction curve of modified sludge soil.

At least five samples were selected for each dosage in the compaction test, and sufficient compaction data were distributed on both sides of the maximum dry density to obtain the compaction curve shown in Figure 9. The compaction parameters of modified sludge soil samples were obtained through curve fitting. The existing fitting methods mainly include the numerical method and curve fitting method. Comparison shows that using a cubic curve to fit the compaction curve is the closest, and higher frequency results in overfitting between the curve and the data. The compaction test data were fitted to obtain the optimal moisture content and maximum dry density, as shown in Table 1. The variation in maximum dry density and optimal moisture content with dosage is shown in Figure 9.

(1) The variation law of compaction curve with dosage

From Figure 9, it can be seen that when the moisture content is below the optimal value, the dry density increases with the increase in moisture content because the water film acts as a lubricant. After exceeding the optimal moisture content, the dry density decreases because the water film offsets the compaction work. The compaction curve of the modified sludge soil exhibits a unimodal characteristic. With the increase in modifier, the compaction curve shifts to the right, and the shape becomes gentle on the left and steep on the right. Compared with untreated soil, modified sludge soil has a narrower range of moisture content changes, reduced plasticity, and increased sensitivity to moisture content. This may be due to the aggregation of small particles into larger ones, which increases frictional resistance, while the water film bonding weakens, requiring a higher moisture content for lubrication.

(2) The variation law of maximum dry density with the dosage of modifier

**Figure 10:**
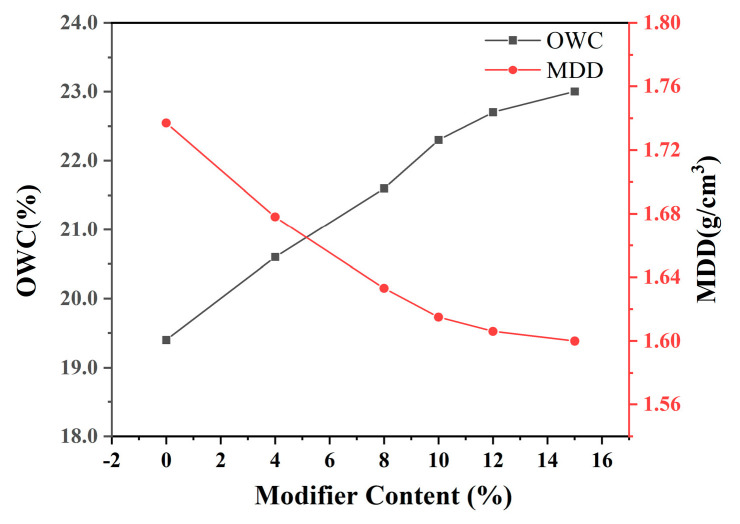
Maximum dry density, optimal moisture content vs. modifier content.

As shown in Figure 10, with the increase in modifier dosage, the maximum dry density of modified sludge soil decreases. For example, when the dosage is 15%, the maximum dry density decreases from 1.737 g/cm^3^ to 1.600 g/cm^3^. This is because the active minerals in the modifier react with the sludge to form a lightweight and bulky crystal structure, causing small particles to agglomerate into larger particles and form aggregates, thereby reducing compaction performance.

(3) The variation law of optimal moisture content with the dosage of modifier

From the compaction curve in Figure 10, it can be seen that the optimal moisture content of modified sludge soil increases with the increase in modifier dosage. Within the range of pure soil to 15% content, the optimal moisture content increases from 19.4% to 23.0%, with the growth rate initially fast and then slow, and the increase slows down after 10% content. This indicates that the modifier causes soil particles to aggregate, increasing the water and work required for compaction, as the modifier reduces the water film on the surface of the clay particles and requires more water lubrication.

### 3.2. Results and Analysis of Unconfined Compressive Strength Test

**Figure 11:**
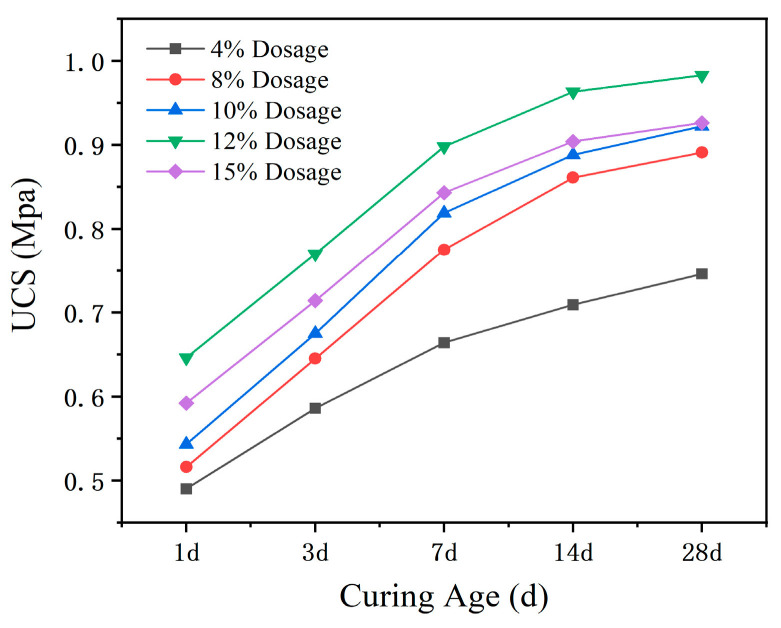
Unconfined compressive strength vs. modifier dosage.

The compressive strength is shown in Table 2, and Figure 11 is used to analyze the variation in the compressive strength of modified sludge soil with the increase in modifier dosage during different curing ages.

From Figure 11, the following can be seen:

(1) The dosage of modifier significantly improved the unconfined compressive strength of modified sludge soil. For example, the 1-day compressive strength of 12% modified sludge soil is 0.676 MPa, which is 37.9% higher than that of 0.49 MPa with 4% content. This improvement is similar in the 7 day and 14 day maintenance periods. Overall, the compressive strength of modified sludge soil is significantly increased compared to silt plain soil.

(2) SM-C type modifier can significantly improve the compressive strength of modified sludge soil when the dosage is less than 12%, but after exceeding 12%, the strength growth slows down or even decreases. This may be due to excessive hydration products blocking the pores, hindering further hydration reactions, and an uneven distribution of modifiers in the sludge. Therefore, it is necessary to determine the optimal dosage through indoor testing to avoid a decrease in strength.

**Figure 12:**
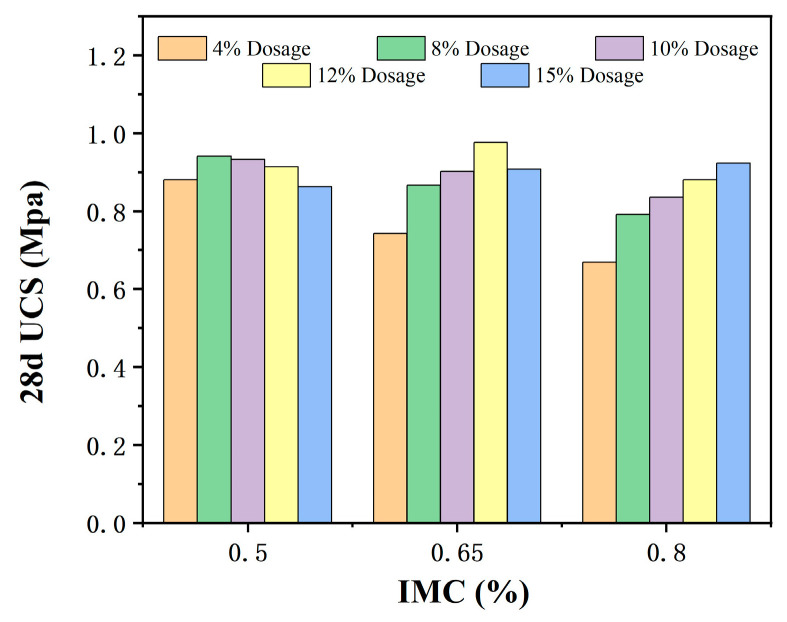
Modifier dosage vs. 28 day UCS value.

Figure 12 shows the effect of different initial moisture contents on the unconfined compressive strength and modifier dosage of modified sludge soil over a 28 day curing period. Due to differences in the mineral composition and the formation environment of sludge, the initial moisture content varies greatly. Usually, the higher the initial moisture content, the more modifier dosage is required to achieve optimal results.

At a fixed dosage of modifier, the initial strength of modified sludge soil with low initial moisture content is higher. However, at the optimal dosage, the strength of different initial moisture contents tends to be consistent. This is because the dosage of the modifier is calculated based on the dry soil mass, and the strength mainly depends on the chemical reaction between the modifier and the silt components. At the optimal dosage, the reaction is sufficient, and the strength improvement is similar.

### 3.3. Results and Analysis of CBR Test

**Figure 13:**
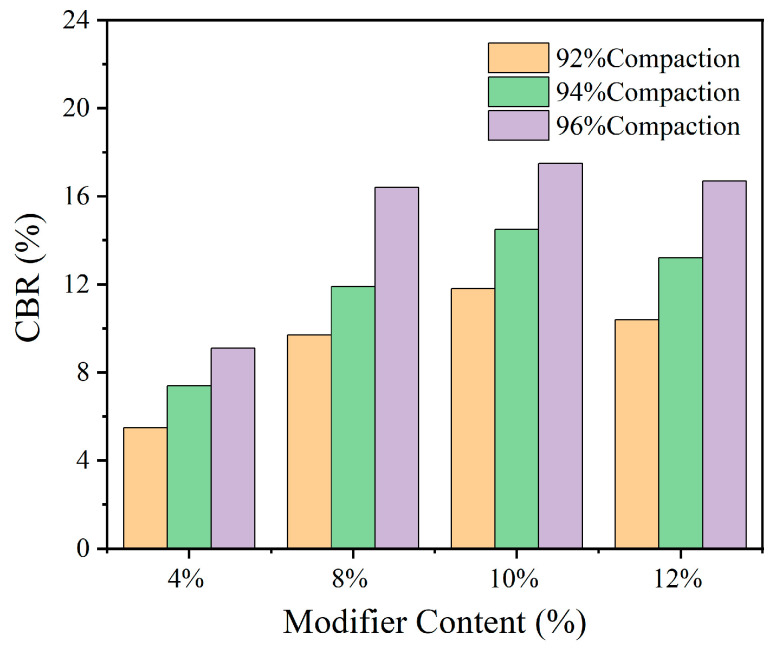
Modifier dosage vs. CBR value.

The CBR test data are shown in Table 3, and Figure 13 is used to analyze the variation in CBR with the change in modifier dosage under different compaction degrees. Referring to the results of the unconfined compressive strength test, the initial moisture content is set at 65%. Within 7 days of curing, the strength has basically formed. Therefore, 7 days are selected as the curing age for the CBR test.

From Table 3 and Figure 13, the following can be concluded: The CBR value of modified sludge soil significantly increased within 7 days, and increased rapidly and then slowly with the dosage of modifier, reaching its peak at 10% dosage. Afterwards, due to an uneven distribution of modifiers, the CBR value decreased. The CBR value also increases with the increase in compaction degree. For roads in low traffic scenic areas, the modified sludge soil treated with SM-C modifier can meet the technical requirements, and the effect of compaction degree on the improvement of the CBR value is more significant under high dosage.

### 3.4. Results and Analysis of Triaxial Test

#### 3.4.1. Analysis of Test Results

The range of variation in dosage from 4% to 12%, and the difference in the principal stress of the specimens under different confining pressure conditions are shown in Table 4. The maximum principal stress difference is taken as the peak stress in the stress–strain relationship, and the strain softening relationship without peak stress is taken as the stress difference corresponding to a strain value of 15%. According to the data in the table, the dosage of modifier significantly improves the shear strength of the silt soil sample.

The shear strength parameters of the modified sludge soil are calculated by taking the common tangent of the Mohr circle. Under three different confining pressure conditions, corresponding to three Mohr circles, the diameter of the Mohr circle is the corresponding difference in principal stress. The calculated shear strength parameters of the soil sample are shown in Table 5. From the data in the table, it can be seen that with the increase in dosage, the internal friction angle and cohesion of the modified sludge soil are significantly improved, and the improvement rate shows a trend of first fast and then slow. When the dosage is below 10%, the internal friction angle increases significantly, mainly manifested by the agglomeration of small soil particles into larger particles, and more obvious interlocking between soil particles. When the dosage is high, the cohesion increases significantly, manifested microscopically as the bonding effect of hydration products.

#### 3.4.2. Stress–Strain Analysis

**Figure 14:**
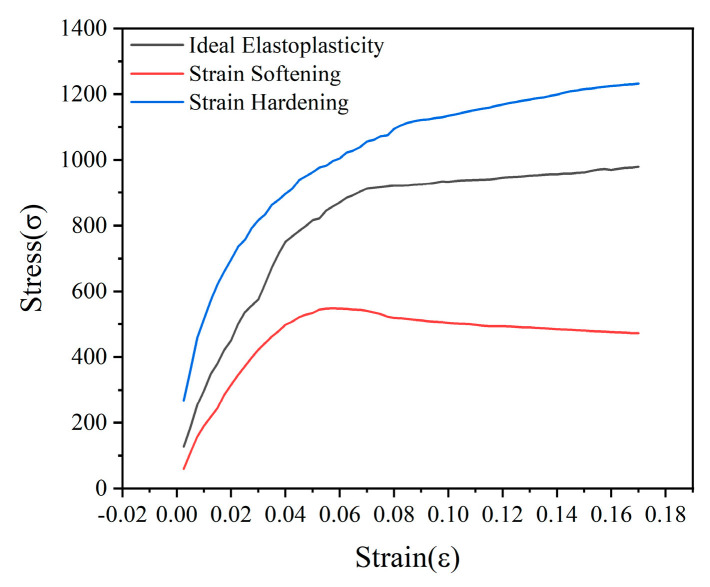
Basic types of stress–strain relationships in consolidation undrained test.

Figure 14 shows three basic types of stress–strain relationships: strain hardening type, ideal elastoplastic type, and strain softening type. These types are closely related to the stress state and internal structure of the specimen, influenced by factors such as the amount of modifier and confining pressure. At the same dosage, as the confining pressure increases, the specimen tends towards the strain hardening type; under the same confining pressure, as the dosage increases, the specimen tends towards the strain softening type.

**Figure 15:**
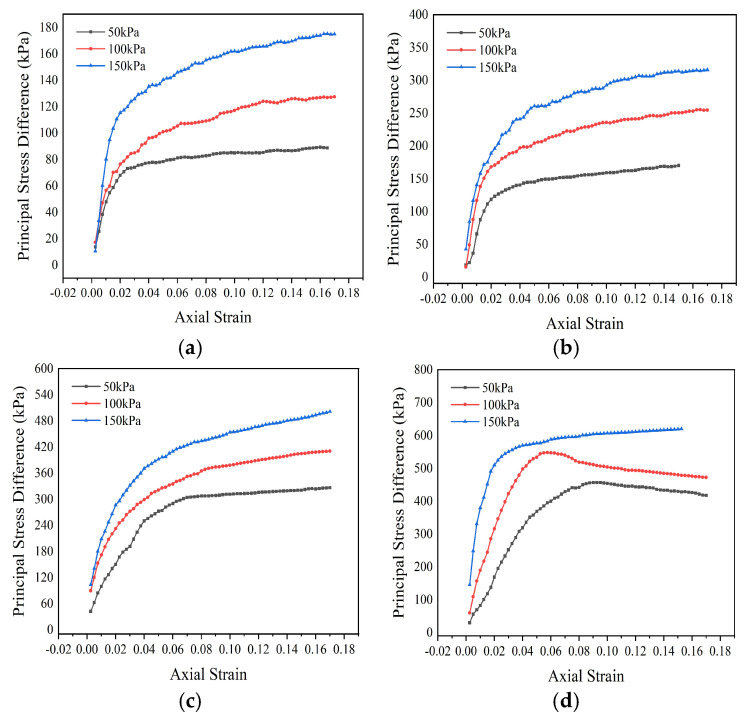
Modifier dosage vs. stress–strain relationship (consolidation undrained test): (**a**) 4%; (**b**) 8%; (**c**) 10%; (**d**) 12%.

Figure 15 shows the stress–strain behavior of consolidated undrained specimens under different dosages of modifiers. In the initial stage, the stress–strain relationship is approximately linear, and the specimen undergoes elastic deformation; subsequently, plastic strain appeared, with a non-linear relationship, and the specimen yielded; finally, the stress growth slows down, the specimen hardens, and the stress stabilizes. When the dosage of modifier increases, the transition point from elasticity to yield of the specimen shifts to the left, and the maximum difference in principal stress increases, indicating that the increase in dosage enhances the shear strength of the specimen.

The 4% modified sludge soil specimen has more pores and less hydration reaction, resulting in low stress and large plastic strain, which may cause mudification in areas with low compaction. The specimens with a dosage of 8% to 12% are close to the optimal dosage, which improves the shear strength, fills the pores with hydration products, enhances the cohesion between soil particles, and forms a stable soil skeleton support.

**Figure 16:**
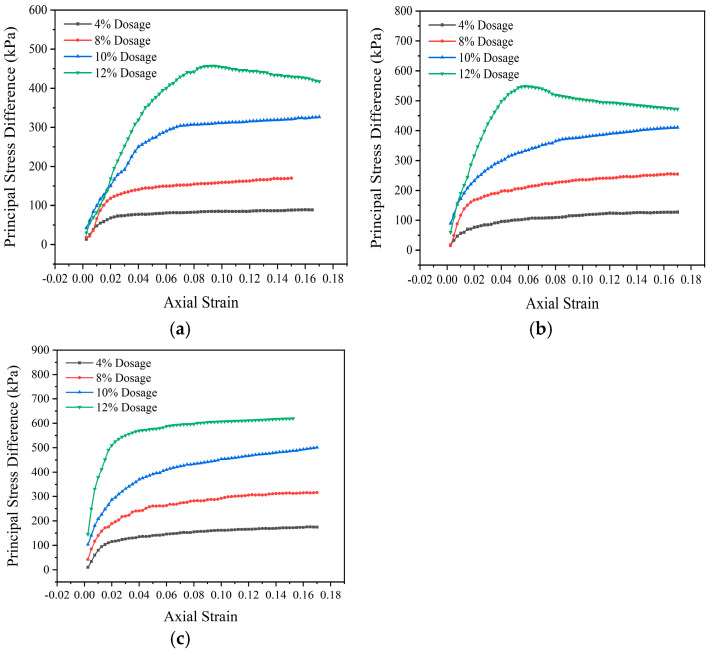
Stress–strain relationship of specimens under different confining pressures: (**a**) 50 kPa; (**b**) 100 kPa; (**c**) 150 kPa.

Figure 16 shows that under low confining pressure, the stress–strain relationship of modified sludge soil changes from the hardening type to the softening type, while under high confining pressure, it tends towards the weak hardening or elastoplastic type. This is because the hydration products generated by the modifier bind the soil particles and fill the pores, enhancing friction. As the confining pressure increases, high content soil can produce sufficient hydration products, which not only bond and fill but also provide skeleton support, leading to brittle failure; low content soil, on the other hand, maintains plastic failure.

### 3.5. Subsection

The compression modulus and compression coefficient are both parameters that describe the compression performance of soil. The compression coefficient is defined as the slope of the tangent line of a certain pressure segment on the e-p curve obtained from the compression test, and the compression modulus is defined as the ratio of stress to strain under the complete confinement condition of the consolidation test. The compression modulus values vary greatly in different load intervals, so the compression modulus is taken as the vertical pressure range of 100 kPa~200 kPa. The same vertical pressure is convenient for comparing the compression performance. According to the specifications, the compression coefficient and compression modulus were calculated, and the test results are shown in Table 6.

Table 6 shows that the compressibility of modified sludge soil decreases with different amounts of modifiers, manifested as a decrease in compression coefficient and an increase in compression modulus. Compared with the original silt, the compression coefficient of the modified soil is lower than 0.5 MPa^−1^, indicating that it has transformed from a highly compressible soil to a moderately compressible soil. When the dosage is 4%, the compressibility is still high because there are many pores in the soil and the skeleton is not sufficient to resist compression. As the dosage increases, the compression performance improves because hydration products fill the pores, form a skeleton, and enhance the anti-compression ability.

**Figure 17:**
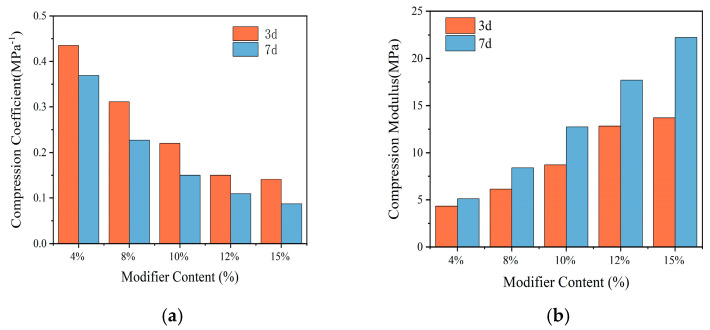
Modifier dosage vs. compressive properties: (**a**) the influence on compression coefficient; (**b**) the influence on compression modulus.

From Figure 17, it can be analyzed that with the increase in modifier dosage, the compression coefficient of modified sludge soil decreases sharply, and the decrease slows down after the dosage reaches 10%, indicating a turning point. Under pressures ranging from 100 kPa to 200 kPa, the compression deformation of the specimens decreases, indicating that the main consolidation stage is nearing completion. The compression coefficients of the specimens with 12% and 15% dosages are similar. When the dosage is between 4% and 10%, the compression coefficient decreases linearly, indicating a rapid improvement in the anti-compression performance. The increase in maintenance age also improves the compression coefficient as the continuous hydration reaction generates products that fill the pores, reduce moisture, further reduce compression deformation, and enhance compression performance.

**Figure 18:**
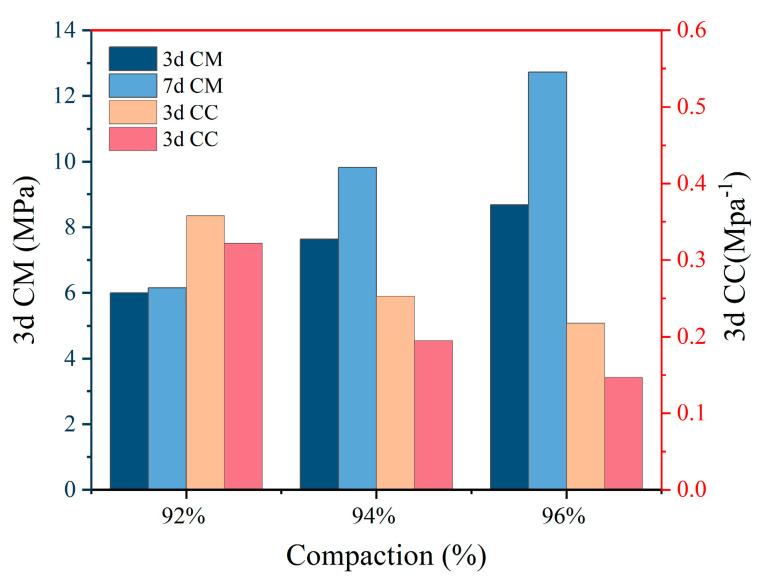
Compaction degree vs. compression performance.

Figure 18 shows that the compression coefficient of modified sludge soil decreases with increasing compaction degree. The addition of modifiers quickly enhances the strength and initial compressive resistance of modified sludge soil. But as the curing age increases, the hydration reaction slows down, and the compression deformation of low-pressure solid specimens is large, while the compression performance of high-pressure solid specimens is improved more significantly due to a denser skeleton.

## 4. Conclusions

This article uses indoor experiments to study the mechanical properties of modified sludge soil, and the main research conclusions are as follows:

(1) The compaction test of modified sludge soil shows that increasing the dosage of modifier will increase the optimal moisture content, reduce the maximum dry density, and cause soil particles to aggregate, increasing the required compaction work.

(2) The unconfined compressive strength test shows that with the increase in modifier dosage and age, the compressive strength first increases rapidly and then slowly, and there is an optimal dosage, beyond which the strength decreases.

(3) According to the CBR test of modified sludge soil, as the dosage and compaction degree increase, the CBR of modified sludge soil significantly improves. When the dosage reaches 8%, it exceeds the minimum specification requirement for CBR. The dosage is the main influencing factor of CBR, and when the dosage is high, the impact of the compaction degree on the CBR of modified sludge soil increases.

(4) According to the triaxial test, the internal friction angle and cohesion of modified sludge soil increase with the increase in dosage. The main failure mode of triaxial testing is plastic failure, and the stress–strain relationship mainly presents as the strain hardening type. With the increase in dosage, the strain hardening type transforms into the strain softening type.

(5) According to the consolidation test of modified sludge soil, with the increase in dosage and curing age, the compression coefficient of modified sludge soil decreases, and the compression modulus increases. At high dosage, the specimen basically shows low compressibility, and the overall compression energy absorption is significantly improved.

(6) Considering that the mechanical properties of improved sludge soil vary over time, future research can focus on long-term performance evaluation, including durability, aging effects, and stability under different environmental conditions.

## Figures and Tables

**Table 1:** Specific compaction parameters of modified sludge soil.

Modifier Content (%)	0	4	8	10	12	15
OWC (%)	19.4	20.6	21.6	22.3	22.7	23
MDD (g/cm^3^)	1.737	1.678	1.633	1.615	1.606	1.600

**Table 2:** Results of Specific compaction parameters of modified sludge soil.

Curing Age	Unconfined Compressive Strength Value (MPa)				
	4%	8%	10%	12%	15%
1d	0.490	0.516	0.543	0.646	0.592
3d	0.586	0.645	0.675	0.770	0.714
7d	0.664	0.775	0.819	0.898	0.843
14d	0.709	0.861	0.888	0.963	0.904
28d	0.746	0.891	0.922	0.983	0.926

**Table 3:** CBR test results of modified sludge soil.

Degree of Compaction	CBR Value			
	4%	8%	10%	12%
92%	5.5	9.7	11.8	10.4
94%	7.4	11.9	14.5	13.2
96%	9.1	16.4	17.5	16.7

**Table 4:** Difference in principal stress of triaxial specimens under different confining pressures (kPa).

Confining Pressure (kPa)	Principal Stress Difference (kPa)			
	4%	8%	10%	12%
50	88.13	170.01	226.33	273.96
100	124.87	237.87	303.67	394.45
150	172.02	297.96	374.00	476.36

**Table 5:** Shear Strength Parameters of Sludge Modified Soil.

4%		8%		10%		12%	
φ (°)	c (kPa)	φ (°)	c (kPa)	φ (°)	c (kPa)	φ (°)	c (kPa)
17	18	24	41	27	78	30	91

**Table 6:** Compressive coefficient and modulus of modified sludge soil.

Curing Age	a_1-2_ (MPa^−1^)					Es_1-2_ (MPa)				
	4%	8%	10%	12%	15%	4%	8%	10%	12%	15%
3 d	0.586	0.645	0.675	0.770	0.714	0.586	0.645	0.675	0.770	0.714
7 d	0.746	0.891	0.922	0.983	0.926	0.746	0.891	0.922	0.983	0.926

## Data Availability

The original contributions presented in the study are included in the article. Further inquiries can be directed to the corresponding author.
